# Supplementary material for: Content-rich biological network constructed by mining PubMed abstracts
Source: BMC Bioinformatics. 2004 Oct 8;5:147. doi: 10.1186/1471-2105-5-147 (PMC528731; doi:10.1186/1471-2105-5-147)
Supplement: Additional File 2 — The original results of the above study (non-essential files are deleted to keep the file size under the limit set by BMC bioinformatics). [file 1471-2105-5-147-S2.bz2 › chilibotAdditionalFile2/dip30/367ID9045692E6041/html/FGF2_FGFR3.html]

 


 **FGF2** and **FGFR3** 
  
Found 77 abstracts in PubMed, retrieved 30.  
 

 What does Google say? 
 PDF only 
| .edu only 

---

**Interactive relationship** (e.g. stimulation, inhibition, etc)

**Neutral relationship**- **FGFR3**  preferentially mediates the mitogenic response to FGF1 and poor response to  **FGF2** .  Ref: 11134040 J Biol Chem, 2001
- Sf9 cells transfected with the corresponding cDNA express the soluble form of  **FGFR3**  AT III into the condition medium and its secreted form was able to bind both FGF 1 and FGF 2  [ **FGF2** ]  leading to loss of ligand binding specificity.  Ref: 11906172 Biochem Biophys Res Commun, 2002
- With some exceptions, FGF 2  [ **FGF2** ]  and the receptor FGFR2, were found in the cytoplasm and occasionally in the nuclei of cells of odontogenic epithelium, while FGF 1 and the receptor  **FGFR3** , were absent or only focally or weakly detected, using standard immunohistochemical techniques.  Ref: 11488421 J Oral Pathol Med, 2001
- The biological significance of VEGF, EGF, and bFGF  [ **FGF2** ]  is mediated by their receptors, which belong to the family of tyrosine kinase receptors Flt 1 VEGFR 1, KDR VEGFR 2, EGFR, FGFR 1, FGFR 2, FGFR 3  [ **FGFR3** ] , and FGFR 4.  Ref: 12500263 Microsc Res Tech, 2003

**Non-interactive relationship** (e.g. studied together, co-existance, homology, etc.)

- ... FGF 2  [ **FGF2** ]  and  **FGFR3**  mRNAs were up regulated in spinal ganglia.  Ref: 11331533 J Comp Neurol, 2001
- The objective of this study was to determine whether FGF 1, FGF 2  [ **FGF2** ] , and high affinity receptors FGFR2 and  **FGFR3**  are present in the pathogenesis of oral epithelial dysplasias and oral squamous cell carcinoma.  Ref: 12075207 Oral Surg Oral Med Oral Pathol Oral Radiol Endod, 2002
- Immunohistochemical localization of growth factors fibroblast growth factor 1 and fibroblast growth factor 2  [ **FGF2** ]  and receptors fibroblast growth factor receptor 2 and fibroblast growth factor receptor 3  [ **FGFR3** ]  in normal oral epithelium, epithelial dysplasias, and squamous cell carcinoma.  Ref: 12075207 Oral Surg Oral Med Oral Pathol Oral Radiol Endod, 2002
- Immunohistochemical localization of fibroblast growth factors FGF 1 and FGF 2  [ **FGF2** ] , and receptors FGFR2 and  **FGFR3**  in the epithelium of human odontogenic cysts and tumors.  Ref: 11488421 J Oral Pathol Med, 2001
- In this work, the bFGF  [ **FGF2** ]  receptors FGFR1, FGFR2 IIIc and  **FGFR3**  genes expression was evaluated to study the correlation between the expression of bFGF  [ **FGF2** ]  receptors and induction of BPH.  Ref: 11517011 Eur J Endocrinol, 2001
- STUDY DESIGN Sections from formalin fixed, paraffin embedded samples of oral normal mucosa n = 14, epithelial dysplasia n = 20, carcinoma in situ n = 10, and squamous cell carcinoma n = 12 were tested for cytoplasmic staining by standard in situ immunohistochemistry with antibodies for FGF 1, FGF 2  [ **FGF2** ] , FGFR2, and  **FGFR3** .  Ref: 12075207 Oral Surg Oral Med Oral Pathol Oral Radiol Endod, 2002
- In complementary in vitro assays, bFGF  [ **FGF2** ]  did not directly stimulate avian OC development from bone marrow mononuclear cell precursors, consistent with their low mRNA expression of the four avian signaling FGF receptors FGFR 1, FGFR 2, FGFR 3  [ **FGFR3** ] , and FGFR like embryonic kinase FREK.  Ref: 12369790 J Bone Miner Res, 2002
- Immunoreactivity for receptors R for IGF 1 and FGF 2  [ **FGF2** ]  IGF 1R, FGFR1, and  **FGFR3**  was most pronounced in chondroblasts and hypertrophic chondrocytes, while FGFR2 immunoreactivity was strongest in the articular and prechondroblastic zones.  Ref: 12243968 Arch Oral Biol, 2002
- The effects of increased  **FGFR3**  activation were determined by exposing cochlear explants to  **FGF2** , a strong ligand for several FGF receptors.  Ref: 12417662 J Neurosci, 2002
- In order to investigate which receptor s may mediate  **FGF2**  signaling and their role in regeneration, we have studied expression of the four fibroblast growth factor receptors, FGFR1, FGFR2,  **FGFR3**  and FGFR4, and of the spliced variants, sFGFR and KGFR, in the regenerating spinal cord of the adult urodele, Pleurodeles waltl, following tail amputation.  Ref: 12379240 Neuroscience, 2002
- Using semiquantitative reverse transcription polymerase chain reaction, we examined the expression of insulin like growth factor 1 Igf 1, fibroblast growth factor 2  [ **FGF2** ]  Fgf 2  [ **FGF2** ] , and their receptors Igf 1r, Fgfr1, Fgfr2, and  **Fgfr3**  in the MCC of 28 day old rats at, and 14 days after placing intraoral appliances designed to produce a lateral functional shift of the mandible.  Ref: 12594422 Am J Orthod Dentofacial Orthop, 2003
